# Tribological and Mechanical Behaviors of Polyamide 6/Glass Fiber Composite Filled with Various Solid Lubricants

**DOI:** 10.1155/2013/320837

**Published:** 2013-05-26

**Authors:** Duxin Li, Ying Xie, Wenjuan Li, Yilan You, Xin Deng

**Affiliations:** ^1^State Key Laboratory of Powder Metallurgy, Central South University, Changsha 410083, China; ^2^Central South University of Forestry and Technology, Changsha 410083, China

## Abstract

The effects of polytetrafluoroethylene (PTFE), graphite, ultrahigh molecular weight polyethylene (UHMWPE), and their compounds on mechanical and tribological properties of glass-fiber-reinforced polyamide 6 (PA6/GF) were studied. The polymeric materials were blended using twin-screw extruder and subsequently injection molded for test samples. Mechanical properties were investigated in terms of hardness, tensile strength, and impact strength. Friction and wear experiments were run under ambient conditions at a rotating speed of 200 rpm and load of 100 N. The morphologies of the worn surfaces were also observed with scanning electron microscope. The results showed that graphite could increase the tensile strength of PA6/GF-15 composite, but the material became soft. Graphite/UHMWPE complex solid lubricants were effective in increasing the already high impact strength of PA6/GF-15 composite. 5% PTFE gave the maximum reduction in the coefficient of friction. However, PTFE/UHMWPE complex solid lubricants were the best choice for improving both friction and wear behaviors due to the lower friction coefficient and mass wear rate. Moreover, the worn surface of PA6 composites revealed that adhesive wear, abrasive wear, and fatigue wear occurred in this study.

## 1. Introduction

In recent years, polymer composites have been widely used for tribological applications to replace traditional metallic materials, which made the studies of friction and wear behaviors of these materials a commercial necessity [1, 2]. As one important engineering plastic, polyamide 6 (PA6) is well known for its high strength, excellent corrosion resistance, suitable wear resistance, and favorable self-lubricating property. However, further improvements are still required to meet more demanding applications due to some drawbacks of pure PA6 as a kind of sliding materials, such as high coefficient of friction (COF) under dry sliding and high wear rate and instability at high load conditions, which limit its applications in a wet and low-temperature environment [3–5].

Depending on their application areas, reinforcement materials such as glass and carbon, particularly in the form of fibers, have been used to enhance the mechanical properties of polymers and reduce cost when compared to the materials of similar strength [6, 7]. When filled with glass fiber (GF), superior properties including tensile strength, hardness, impact strength, and dimensional stability of PA6 can be changed effectively. This is generally good for tribological properties as well [8]. GF-reinforced PA6 has been producing in large quantities, such as PA6/GF (15 wt%).

It has been known for quite some time that solid lubricant exhibits a self-lubricating behavior and its application in sliding prevents stick-slip motion instabilities [9]. It is thus used as an additive into polymers to obtain good friction and wear properties. The studies on the friction and wear of polymer composites filling such solid lubricants as polytetrafluoroethylene (PTFE), graphite, and ultrahigh molecular weight polyethylene (UHMWPE), and others have reported that solid lubricant reduces the coefficient of friction and wear rate of some composites but increases the wear rate of others [10–12]. This makes it imperative to evaluate the effect of its addition to new materials or composites. Besides, in most articles, people normally selected single solid lubricant as the filler. However, based on our previous work [13], it has been proved more effective to choose PTFE and graphite as complex solid lubricants to improve mechanical and tribological properties of PA6. With the application of complex solid lubricants, synergistic reaction can be gained to modify the tribology capabilities of PA6. Hence, it is worth to make further efforts.

PTFE is one of the generally used solid lubricants that could reduce the coefficient of friction and, due to this fact, sometimes the wear rate of polymeric composites [14]. As one of the three forms of carbon, graphite has a layer structure in which the carbon atoms are arranged in a hexagonal unit cell within each layer. These layers are linked by weak Van der Waals bonds, which may be easily broken by shear forces under sliding conditions. Therefore, graphite is also a potential candidate as filler, which could form a transfer film on the sliding counterpart [15]. UHMWPE possesses excellent self-lubrication properties and high impact resistance and chemical stability, which make it widely used in engineering applications [16]. 

With the aim of improving the mechanical and tribological properties of PA6 further and based on the above observations, it was decided to reinforce PA6 with glass fiber firstly and then fill it with single PTFE, graphite, UHMWPE; and their complex solid lubricants. As a new kind of material, GF reinforcement and solid lubricants could be expected to significantly improve the mechanical and tribological behaviors of PA6 and offer strong potential in the field of lubricated sliding application. In fact, some limited studies investigating the effects of single solid lubricants on the performance of pure PA6 have been made. However, the research results on the friction and wear properties of PA6/GF composites are rarely found in the literature, especially for effect of complex solid lubricants. The present work would focus on the topic to clarify the effects of single and complex solid lubricants on the mechanical and tribological behaviors of PA6/GF composites.

## 2. Experimental

### 2.1. Materials and Specimens Preparation

PA6 and PA6/GF used in this experiment were produced by Shenzhen Changtai Plastic Co. Ltd. (BST 425, Shenzhen, China). PTFE (A-Grade) was purchased from Tangshan Rifeng Chemical Co. Ltd. (Tangshan, China). Graphite with a particle size of 25 *μ*m was purchased from Shanghai Yifan graphite Co. Ltd. (Shanghai, China). UHMWPE was purchased from Shanxi Zhongke TianGang Technology Development (Lvliang, China).

Prior to blending, PA6 and PA6/GF were dried at 80°C for 8 h in vacuum oven to remove most of absorbed humidity. The composites were prepared by twin-screw extruder. The extruder had nine heating zones, and the temperature profiles of the barrel were 210-220-230-240-240-240-240-240-240°C from the hopper to the die. The screw frequency and the feeding frequency were set at 360 Hz and 20 Hz, respectively. The extrudate in the form of cylindrical rod was subsequently cooled down in a water bath and then pelletized. In order to ensure steady-state operation and flush impurities out of the system before getting the polyblends samples, the initial 1 kg of the extrudate was discarded.

Then the injection molding technique was applied to prepare the specimens. The injection pressure was 45 MPa. The temperatures in four zones were 250, 250, 250, and 250°C and in the nozzle 230°C. The molded specimens were visually inspected for air bubbles, and those with defects were discarded.

The content of glass fiber in the PA6/GF composite as we received was 15%, marked PA6/GF-15. The single solid lubricant (PTFE, graphite, UHMWPE) weight contents were 5%, 8%, 10%, 12%, 15%, and 20% for each blend. And the total content of complex solid lubricants (PTFE/graphite, PTFE/UHMWPE, and graphite/UHMWPE) was fixed at 15% with the composition ratio of 3/12, 5/10, 7/8, 10/5, and 12/3.

### 2.2. Characterization

Tensile test was performed using CMT-7205 Universal Electronic Test Machine (Zhuhai, China) at a beam rate of 5 mm/min at room temperature. Samples were cut into narrow-waisted dumbbell-shaped specimens in accordance with the Chinese standard GB/T1040-2006. 

Impact fracture tests to the simple beams with “U” notch at one side of specimen are implemented on a JB-S Impact Test Machine (Chengde, China) at room temperature in accordance with the Chinese standard GB/T1043-93. The size of notch is 3.50 × 10.36 mm^2^. Five samples for each material were tested and averaged as the data.

Hardness tests of PA6 composites were carried out on a HD1-1876 Brinell hardness tester (Shanghai, China). A load of 31.25 Kg and loading time of 30 s were used. 

Friction and wear tests were conducted using a reciprocating UMT-3 tribometer (CERT company, USA) at room temperature under dry friction condition. The specimens (25 mm length, 25 mm width, and 10 mm thickness) were cleaned with acetone by ultrasonic bath before testing. The tests were conducted at load of 100 N. The test duration was 2 h and the rotational speed was set at 200 rpm. After the tests, the mass loss was weighed with an analytical balance (accuracy 10^−4^ g) to calculate the specific wear rate by the equation

(1) $$\begin{array}{c} Kw=\frac{\Delta m}{\rho Ld}\;\text{c}{\text{m}}^{3}\;{\text{N}}^{-1}\;{\text{m}}^{-1}, \end{array}$$

where Δ*m* (g) is the mass loss of the specimen, *ρ* (g/cm^3^) is the density of the test material, *L* (N) is the load, and *d* (m) is the total sliding distance. Three samples for each material were tested and averaged as the data.

The worn surfaces of PA6 composites were coated with a thin layer of gold and observed by scanning electron microscope (SEM, JSM-6363LV, Japan).

## 3. Results and Discussion

### 3.1. Mechanical Properties

**Figure 1:**
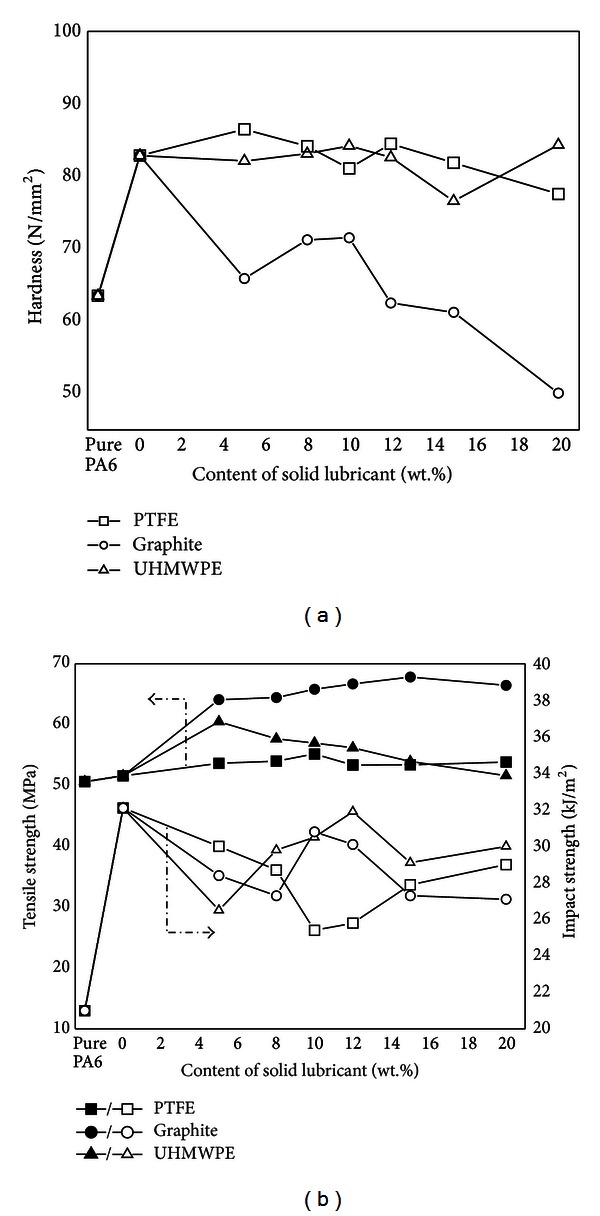
Mechanical property of PA6/GF composites filled with different single solid lubricant (content at zero is PA6/GF-15 sample). (a) Hardness. (b) Tensile strength and impact strength.

**Figure 2:**
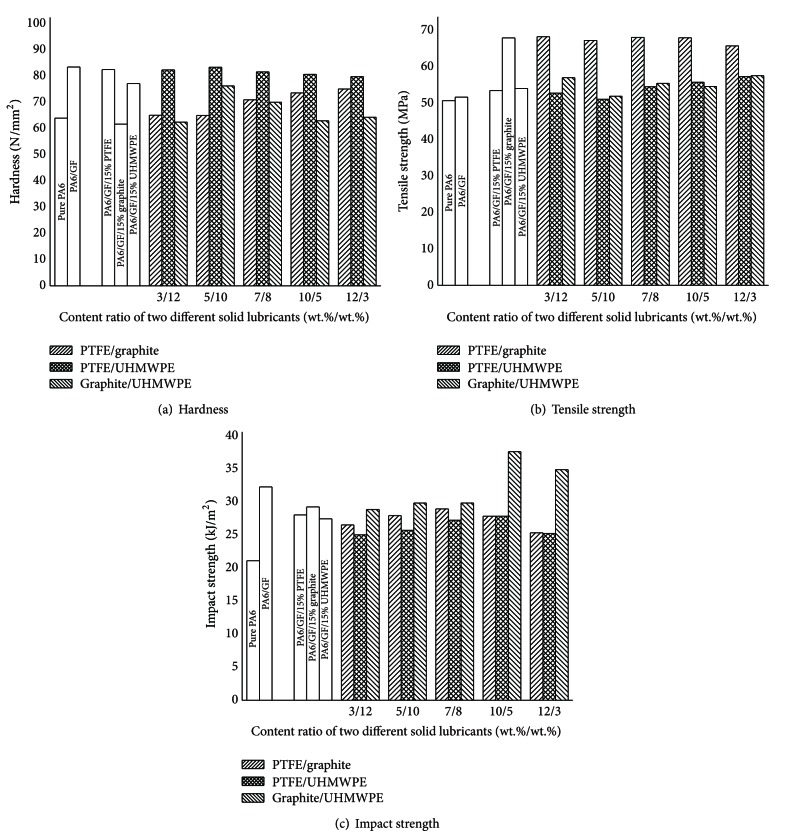
Mechanical property of PA6/GF composites filled with complex solid lubricants.

The effects of single PTFE, graphite, UHMWPE, and their complex solid lubricants on the mechanical properties were studied on PA6/GF-15 composite. And the results were shown in Figures 1 and 2.

From Figure 1(a), it can be found that the hardness of PA6 increased from 63.5 to 82.8 N/mm^2^ by 30% with the addition of 15 wt% GF, which indicated that GF improved the load carrying capacity of PA6. When PTFE and UHMWPE were added to PA6/GF-15 composite individually, there was practically no effect on the hardness of the composites. However, after filled with graphite, the hardness of composite decreased markedly as low as 50 N/mm^2^. This is because graphite is the softest substance as we all know.

When PTFE was added to PA6/GF-15 composite in different proportions, there was a slight increase in tensile strength, as seen in Figure 1(b). Palabiyik and Bahadur reported the similar trend of the tensile strength of PA6 and PA6/PTFE composites [17]. When the composite was filled with graphite, there was 24% increase (51 to 64 MPa) in tensile strength with as little as 5 wt% graphite and the tensile strength increased continuously with increasing content of graphite. This demonstrates that as an inorganic solid lubricant, graphite has good compatibility with GF when added to PA6/GF-15 composite, which facilitated better load transferring to the reinforcing phase and thus improved the strength [18]. It was noticed that the tensile strength increased by 17% when the added amount of UHMWPE was 5 wt%. But then it decreased slowly when adding more UHMWPE. One possible reason is that the interface bonding between PA6 and UHMWPE became weak when the addition of UHMWPE increased over 5 wt%.

Comparing with pure PA6, GF reinforcement increased the impact strength from 21 to 32 kJ/m^2^ by 52%. However, the impact strength decreased when filled with single solid lubricant. Nevertheless, it was still much higher than pure PA6. For PA6/GF-15 composite, when the material suffered shock, GF could absorb more impact energy which resulted in the increase of impact strength [19]. After adding solid lubricant into composite, the contact between GF and solid lubricant increased and GF was probably covered by solid lubricant partially. Thus, the solid lubricants additive would divide the resin structure, which induced stress concentration and the decrease of effective cross-sectional area bearing pressure. As a result, the impact strength of PA6/GF-15 composite decreased. With increasing the content of PTFE, the impact strength decreased first and then increased. The minimum value emerged when the additive amount was 10 wt%. As for graphite and UHMWPE, the impact strength increased first and then decreased in the range of 5~20 wt%. The maximum value appeared at 10 wt% and 12 wt%, respectively. With the addition of UHMWPE, the reduction of impact strength was much lower than filling with PTFE and graphite.

Obviously, GF was an effective reinforcement for PA6 matrix in the present case. Thus, the effects to be noted for the single solid lubricant on mechanical properties of PA6/GF-15 composite are as follows: (1) the effects of PTFE and UHMWPE fillers on hardness and tensile strength are fairly small; (2) graphite solid lubricant increases the tensile strength dramatically but makes the material soft; (3) all the three solid lubricants decrease the impact strength of PA6/GF-15 composite in different degree, which means that single solid lubricant makes the composite material brittle.

Figure 2 illustrated the effects of three kinds of complex solid lubricants, PTFE/graphite, PTFE/UHMWPE, and graphite/UHMWPE, on mechanical properties of PA6/GF-15 composite. The total content of complex solid lubricants was fixed at 15 wt%. As is seen from the hardness in Figure 2(a), when PA6/GF-15 composite was filled without graphite, there was a slight change in hardness, which is the same trend as filling with single solid lubricant. If the softest graphite was added into composite, the hardness decreased.

The variation of the tensile strength of PA6 and its composites with content ratio of complex solid lubricants is shown in Figure 2(b). When PTFE/graphite compound solid lubricant was added to PA6/GF-15 composite, there was approximately 32% increase in tensile strength as good as single graphite with the same amount value of 15 wt%. This is mainly attributed to the presence of graphite particles, which can make the blend structure more homogeneous [13]. Consequently, the stretch-proof capacities of PA6 composites enhanced. However, when graphite and UHMWPE were selected as the compound solid lubricants, there was no obvious increase in tensile strength for composite. This is believed to be caused by the difference of molecular weight between PTFE and UHMWPE, which can influence the compatibility between inorganic graphite material and organic solid lubricants. As for PTFE/UHMWPE compound solid lubricant, there was practically no effect on the tensile strength of PA6/GF-15 composite as the same as single PTFE and UHMWPE solid lubricants.

Figure 2(c) shows the impact strength of PA6 and its composites filled with different complex solid lubricants. It can be noted that with increasing graphite content, the impact strength increased when the content of graphite/UHMWPE compound had a fixed and unchanging value of 15 wt%. And with the proportion 10/5 of graphite and UHMWPE, there was a 16% increase on impact strength. Comparing with the effect of single graphite and UHMWPE additive on impact strength discussed previously, it is mainly attributed to the combination of graphite and UHMWPE. Graphite particles dispersed in PA6 and UHMWPE provided a large surface area available for interaction between the polymer molecules, which enhanced the absorb capacity of impact energy [20]. Besides, when PTFE/graphite and PTFE/UHMWPE were added as complex solid lubricants, there were no big effects on the impact strength of PA6/GF-15 composite.

Therefore, we can infer the conclusions from the facts that graphite was more effective to improve the tensile strength when it was added as single solid lubricant or combined with PTFE. Increasing graphite content increases tensile strength, but it does not always increase impact energy. Yet, combination of graphite and UHMWPE as complex solid lubricants was helpful to improve the impact strength of PA6/GF-15 composite. However, graphite makes the materials much softer. Secondly, the effect of PTFE and/or UHMPWE solid lubricants addition on mechanical properties of PA6/GF-15 composite was negligibly small.

### 3.2. Friction and Wear Behaviors

**Figure 3:**
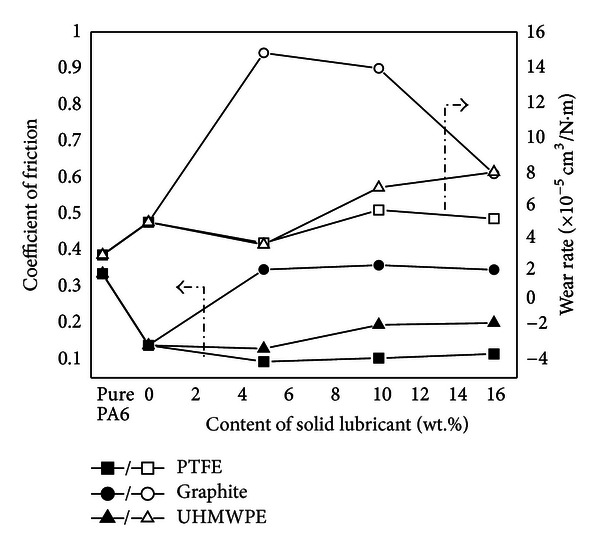
Friction and wear behaviors of PA6/GF composites filled with single solid lubricant.

Figure 3 shows the variation of friction coefficient and wear rate of PA6 and its composites with content of different single solid lubricant under dry sliding. Pure PA6 had a much higher coefficient of friction of 0.34 but a lower wear rate of 3.1 × 10^−5^ cm^3^/Nm. Compared to PA6, PA6 composites exhibited a lower value of friction coefficient. PA6/GF-15 composite possessed a low COF of 0.14 with 59% reduction but much higher wear rate of 5 × 10^−5^ cm^3^/Nm.

With the addition of PTFE, the COF of the composite decreased slightly. As it is known that PTFE yields a very low COF by itself, adding PTFE into polymers can obtain composites with lower COF. Furthermore, PTFE can be easily dragged out from the matrix to form a third-body transfer film, which reduces the direct contact between matrix and counterpart [17]. The emergence of the transfer film can give a further reduction for the COF. To be specific, 5 wt% PTFE-filled PA6/GF-15 composite exhibited the lowest COF with 0.09 and wear rate with 3.8 × 10^−5^ cm^3^/Nm. However, with increasing PTFE, the wear rate increased slightly. This result is closely related to the transfer film formation on the counterpart material. With the increase of PTFE, the transfer film is completed gradually. But PTFE itself has a relatively low wear resistance due to its soft nature. Thus, excessive PTFE content is not profitable for the wear resistance.

When filled with graphite, the COF increased back to 0.34 as high as pure PA6. The possible reason is that the inclusion of graphite softened the composites, as discussed in hardness, which enlarged the actual contact area. According to simple adhesion theory of Bowden and Tabor [21], the frictional force is in proportion to the actual contact area. Consequently, the COF increased. For the wear rate, it demonstrated similarly the variation of COF. The wear rate after addition of graphite went up as high as 1.5 × 10^−4^ cm^3^/Nm, increased by one order of magnitude. 

In the case of UHMWPE solid lubricant, it showed the similar trend as filling with PTFE. However, when the addition of UHMWPE is over 5 wt%, the COF and wear rate increased faster than PTFE filler. This is mainly because of the thermodynamic incompatibility between UHMWPE and PA6 matrix. It was worth noticing that the wear rate of composite was decreased to 3.8 × 10^−5^ cm^3^/Nm, as low as PTFE, when the amount of UHMWPE was 5 wt%. Compared to pure PA6, the improvement of the tribology capabilities is due to the reason that nonpolar UHMWPE has a smooth molecular chain structure, so it can form a boundary layer with low shear strength.

Thus, in this work, PTFE was much more effective to improve friction and wear properties of PA6/GF-15 composite. The composites filled with PTFE exhibited both lower COF and wear rate than the others did. As for graphite, it had no positive effect on friction and wear properties. Neither a reduction of the COF nor a decrease of the mass wear rate did occur. Besides, UHMWPE was also effective. For the modification effects of three single solid lubricants on PA6/GF-15 composite, PTFE was the best, UHMWPE second best, and graphite third best.

**Figure 4:**
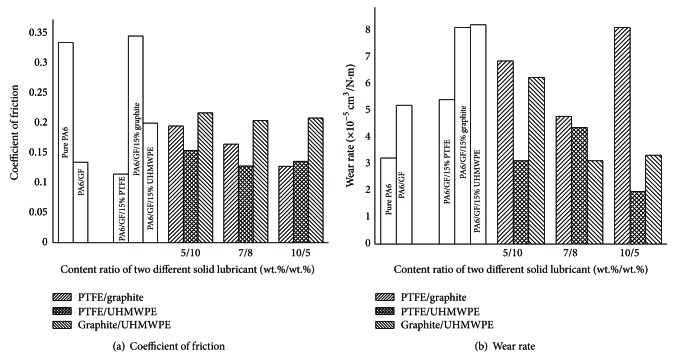
Friction and wear behaviors of PA6/GF composite filled with complex solid lubricants.

In an effort to make further improvements in the tribological behaviors of PA6/GF-15 composite, another possible approach, that was complex solid lubricants, was explored in this work. PA6/GF-15 composite was filled with complex solid lubricants, which were composed of various proportions of PTFE/graphite, PTFE/UHMWPE, and graphite/UHMWPE. The friction and wear behaviors of PA6 and its composites filled with complex solid lubricants were shown in Figure 4.

It was observed from Figure 4(a) that when filled with PTFE and graphite compound, with increasing PTFE content, the COF decreased. When the addition ratio of PTFE and graphite was 10/5, the COF of composite decreased to 0.12 with the reduction of 65% and 14% compared to pure PA6 and PA6/GF-15 composite, respectively. As for PTFE/UHMWPE compound, it showed similar trend with increasing PTFE and the COF decreased also to 0.12 with the addition ratio of 7/8. It is demonstrated from the results above that PTFE was effective for improving the friction properties. When graphite and UHMWPE were combined, the COF increased up to 0.2, which was still lower than pure PA6 but higher than PA6/GF-15 composite. However, the lowest COF value 0.11 was appeared when filled with single PTFE. This is probably due to the compatibility between different solid lubricants.

Figure 4(b) illustrated the effect of complex solid lubricants on the wear rate of the specimens. As discussed above, with the addition of single solid lubricant, the wear rate of composite increased compared to pure PA6 and PA6/GF-15 composite. It is noted that with the addition of PTFE/UHMWPE complex solid lubricants, the wear rate of composites decreased to the lowest 1.9 × 10^−5^ cm^3^/Nm by 39% with the content ratio 10/5 and it was also lower than any single solid lubricant. It was suspected that synergistic reaction existed in the use of PTFE and UHMWPE complex solid lubricants. Besides, graphite is not good for improving wear properties. 

In conclusion, PTFE/graphite and PTFE/UHMWPE complex solid lubricants are helpful for friction properties due to the presence of PTFE. The content of PTFE is crucial. PTFE/UHMWPE compound is the best choice to improve the wear properties because of the synergistic reaction between organic solid lubricants.

### 3.3. SEM Studies on Worn Surface

According to the generally accepted models of material wear, three mechanisms contribute to the wear processes of PA6 composites, which are adhesive wear, abrasive wear, and fatigue wear [22]. Their relative contribution depends on the load level as well as on the chemical, mechanical, and geometrical properties and lubricating conditions.

**Figure 5:**
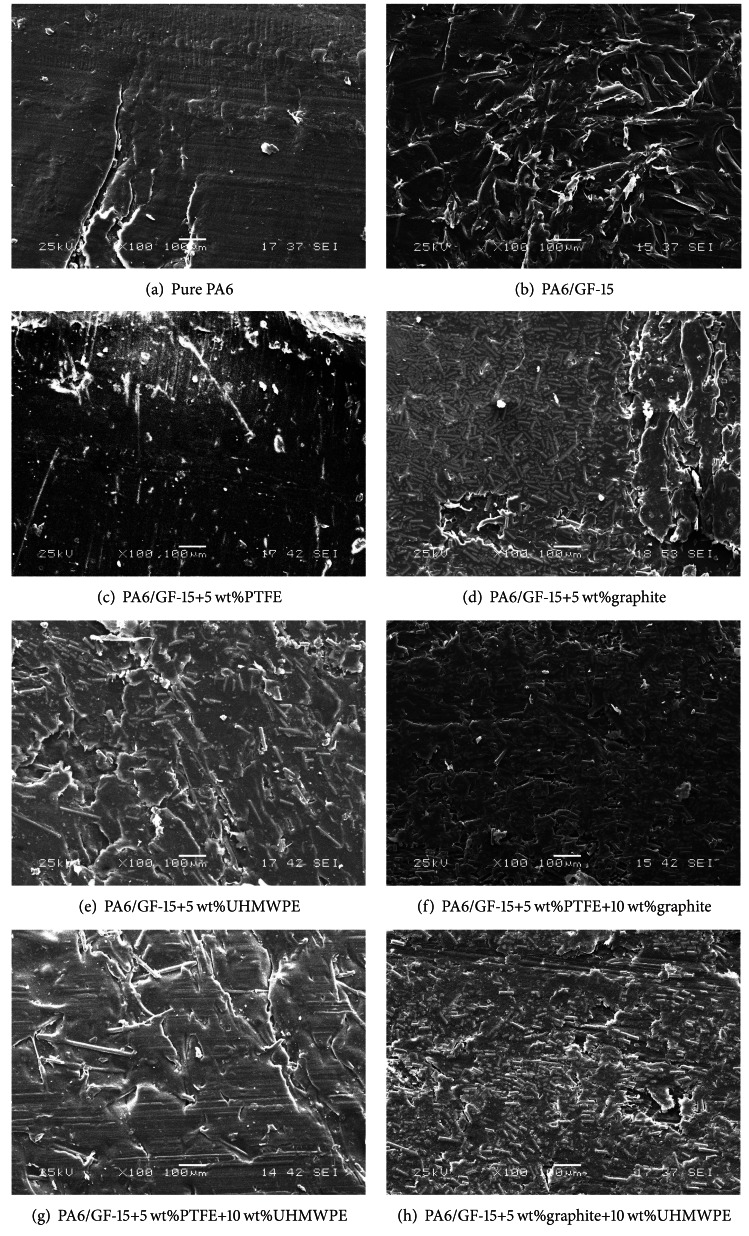
SEM images of worn surface of PA6 composites filled with different solid lubricants.

Figure 5 shows the SEM images of the worn surfaces of PA6 and its composites filled with different single and complex solid lubricants under dry sliding at a normal load of 100 N. As shown in Figure 5(a), the worn surface of PA6 displayed plucked and ploughed marks, which indicated that the wear mechanism was featured with adhesive and plough wear. Due to the increased temperature at two frictional surfaces, severe adhesive wear was mainly attributed to the softening of pure PA6. By contrast, the ploughing and adhesion on the worn surface of PA6/GF-15 composites were considerably reduced from the micrograph in Figure 5(b). It can be seen clearly that some GFs were exposed on the surface of composites. During sliding, these GFs were easily released from the PA6/GF composite to transfer to the contact zone of PA6 composites and the counterfaces. Thus, these GFs could serve as solid lubricant to prevent the direct contact between the two mating surfaces, thereby reducing the friction coefficient and wear rate. As a result, the PA6/GF-15 composites showed much better friction and wear resistance than those of PA6 matrix. 

As for the worn surface of PA6/GF-15 filled with 5 wt% PTFE in Figure 5(c), there existed lamellar structure among the surfaces, due to the plastic deformation. Thus, the main wear mechanism was adhesive wear. And the GF existed on the surface still can be seen. With the addition of graphite, the worn surface in Figure 5(d) differed from those with PTFE. The small graphite particles were dispersed on the surface of composites. When sliding against a steel counterface, the tiny graphite particles in the PA6 matrix played the role of lubricating agent. The bond between graphite and PA6 may prevent graphite particles from being easily transferred onto the surface of counterface. It was also noted that some area on the worn surface was not filled with graphite particles and a little area had peeled off extensively, as the result of fatigue wear. When filled with UHMPWE, it can be seen from Figure 5(e) that there were some lamellar structures shaped in the form of islands, which might be caused by adhesive wear.

From Figure 5(f), a small piece of layer appeared on the worn surface in the case of PA6/GF-15 filled with 5 wt% PTFE and 10 wt% graphite, which was different from single PTFE and graphite fillers. The graphite particles dispersed much better than with the addition of single graphite. So the surface looks more uniform, and the peeling was constrained due to the combination of PTFE and graphite. The main wear mechanism was adhesive wear. The deep rill-like folds can be clearly observed in Figure 5(g), like pure PA6. It is mainly caused by adhesion. Besides, there existed a little crack which was probably induced by abrasive wear. As shown in Figure 5(h), with the addition of 5 wt% graphite and 10 wt% UHMWPE, the graphite particles were not homogeneous as good as single graphite filler. The peeling was constrained compared to single graphite due to the combination of graphite and UHMWPE. Thus, the PA6/GF-15 composite filled with graphite/UHMWPE presented the feature of adhesive wear and fatigue wear.

## 4. Conclusions

In this study, the mechanical and friction and wear behaviors of PA6/GF-15 composite filled with single solid lubricant PTFE, graphite, UHMWPE, and their compounds were comparatively investigated. From the results, the following conclusions can be drawn.

1. Graphite was more effective in improving the tensile strength of PA6/GF-15 composite, but the material became soft. There was, however, no significant effect on the tensile strength and hardness with the addition of PTFE or UHMWPE to the PA6/GF-15 composite. The impact strength of PA6/GF-15 composite decreased when filled with single solid lubricant, but graphite/UHMWPE complex solid lubricants can improve the impact strength of PA6/GF-15 composite by 16%.
2. PTFE was much more effective in improving friction property of PA6/GF-15 composite than graphite and UHMWPE. 5 wt% PTFE as the filler was the most effective. It gave a very low coefficient of friction of 0.09, which is exceptionally low for dry sliding at 100 N. UHMWPE is the second best. Graphite has no positive effect on friction properties. As for the wear property, however, graphite was not helpful in decreasing the wear rate of PA6/GF-15 composite. When added with 5 wt% PTFE and UHMWPE fillers, the wear rate decreased to 3.8 × 10^−5^ cm^3^/Nm. However, with increasing PTFE and UHMWPE, the wear rate increased slightly.
3. Of all the methods used to improve the tribological behavior of PA6/GF-15 composite, PTFE/UHMWPE complex solid lubricants are the best choice for improving both friction and wear properties. When the content ratio was 7/8, it can give a low coefficient of friction of 0.12 and a very low wear rate of 1.9 × 10^−5^ cm^3^/Nm with the reduction of 65% and 14% compared to pure PA6 and PA6/GF-15 composite, respectively.
4. The tribological mechanism of PA6 and its composites are closely related to the varieties of solid lubricants filled. There existed three kinds of wear mechanisms in this work, which are adhesive wear, abrasive wear, and fatigue wear.
